# Basal Metabolic Rate of Adolescent Modern Pentathlon Athletes: Agreement between Indirect Calorimetry and Predictive Equations and the Correlation with Body Parameters

**DOI:** 10.1371/journal.pone.0142859

**Published:** 2015-11-16

**Authors:** Luiz Lannes Loureiro, Sidnei Fonseca, Natalia Gomes Casanova de Oliveira e Castro, Renata Baratta dos Passos, Cristiana Pedrosa Melo Porto, Anna Paola Trindade Rocha Pierucci

**Affiliations:** Department of Nutrition, Federal University of Rio de Janeiro, Rio de Janeiro, Rio de Janeiro, Brazil; University of the Balearic Islands, SPAIN

## Abstract

**Purpose:**

The accurate estimative of energy needs is crucial for an optimal physical performance among athletes and the basal metabolic rate (BMR) equations often are not well adjusted for adolescent athletes requiring the use of specific methods, such as the golden standard indirect calorimetry (IC). Therefore, we had the aim to analyse the agreement between the BMR of adolescents pentathletes measured by IC and estimated by commonly used predictive equations.

**Methods:**

Twenty-eight athletes (17 males and 11 females) were evaluated for BMR, using IC and the predictive equations Harris and Benedict (HB), Cunningham (CUN), Henry and Rees (HR) and FAO/WHO/UNU (FAO). Body composition was obtained using DXA and sexual maturity data were retrieved through validated questionnaires. The correlations among anthropometric variables an IC were analysed by T-student test and ICC, while the agreement between IC and the predictive equations was analysed according to Bland and Altman and by survival-agreement plotting.

**Results:**

The whole sample average BMR measured by IC was significantly different from the estimated by FAO (p<0.05). Adjusting data by gender FAO and HR equations were statistically different from IC (p <0.05) among males, while female differed only for the HR equation (p <0.05).

**Conclusion:**

The FAO equation underestimated athletes’ BMR when compared with IC (T Test). When compared to the golden standard IC, using Bland and Altman, ICC and Survival-Agreement, the equations underestimated the energy needs of adolescent pentathlon athletes up to 300kcal/day. Therefore, they should be used with caution when estimating individual energy requirements in such populations.

## Introduction

Humans total energy expenditure (TEE) is defined by the sum of basal metabolic rate (BMR), thermal effect of food, and the energy expenditure due to physical activity[1]. In adolescents, the TEE is also influenced by the energy expenditure from anabolism/growth[2]. The BMR is the energy required to maintain the physiological processes for absolute rest and fasting status (12 hours) and comprises approximately 70% of TEE in sedentary individuals and 45 to 60% in athletes and physically active individuals.

The use of predictive equations is the most viable method for the BMR estimation of individuals, group or population, as regards aspects such as costs. However, some authors have shown that equations can provide under or overestimated results in BMR when it is applied in an individual level or in different ethnical groups or populations that differ from the original studies that validated such equations[3–7].

The TEE estimative through predictive equations requires multiplying BMR by the physical activity level (PAL) considered adequate for an individual. PAL is a numeric expression of an individual’s daily physical activity, comprising of obligatory and discretionary physical activities exerted throughout a day[8]. Therefore, an adequate PAL estimation for physical activities that comprise of several sport modalities, such as modern pentathlon, is not always so easily predicted. After the election of a PAL value, it must be multiplied by BMR in order to determine TEE. Although there are lots of possible errors in estimating the adequate PAL, an inappropriate BMR estimation will be then multiplied, amplifying the under or over estimative of TEE. Thus, the validity of equations for the prediction of BMR is a subject of great discussion, especially when it comes to peculiar populations, as it is the case of adolescent modern pentathlon athletes. Making an accurate prediction of BMR is crucial for nutritional counselling and promoting proper physical development.

In this context, several authors question the use of equations for BMR estimative, and recommend the use of indirect calorimetry (IC), which presents itself as a non-invasive gold standard method, characterized by safety and convenience. However, IC can be expensive and its applicability on the field as a routine evaluation is not a reality in many sport training centers. Being the use of equations an easier, less expensive.

In endurance sports, the accurate estimation of individual energy needs is necessary to establish appropriate dietary prescriptions, in order to provide athlete’s improved immunity[9], performance[10], as well as reduced susceptibility to injury[11,12], endocrine changes[10] and avoid female athlete triad[13] with menstrual disturbances[14]. Insufficient dietary intake increases the risk of stress fractures in both males and females[15]. Menstrual dysfunction[16], poor bone health[17,18], history of fracture[19] and eating psychopathology[18–20] can also be further risks when inadequate dietary intake is presented.

Given the complexity of Modern Pentathlon—which consists in five different events (fencing, swimming, jumping, and a combination of pistol shooting and cross-country running), the competition can last up to 8 hours, making physical exhaustion almost unavoidable. To date, there are few works available addressing physiological demands of modern pentathlon athletes[21] and we have found no data on the literature that could support energy needs of pentathletes.

Therefore, the aim of this study was to evaluate the BMR of pentathletes by an indirect and scientifically certified method for this purpose, and to examine the agreement of the BMR estimated by most cited predictive equations Harris and Benedict (HB) (1919)[22], Cunningham (CUN)[23], Henry and Rees (HR)[24] and FAO/WHO/UNU (FAO)[8]/Schofield[25] with the BMR measured by IC. Additionally, we aimed to verify the association among anthropometric variables and BMR measured by IC.

## Materials and Methods

### Subject sampling

The sample of our cross-sectional study consisted of 28 athletes—members of the Modern Pentathlon Rio de Janeiro Federation (FPMRJ)—with ages ranging between 11 and 17 years old, being 17 males and 11 females. Athletes participating in this study were subject to the following inclusion criteria: at least 3 hours of training a day, six days a week and practicing this sport for at least one year prior to the sample recruitment. The subject sample corresponded to 96.5% of the universe of the FPMRJ adolescent athletes. Athletes and their parents/legal guardians received a document explaining all aspects of research and the volunteers' participation was subject to the presentation of this consent form signed by both. This research was approved by the Ethical Committee on Researches among Humans subjects of the University Hospital from the Federal University of Rio de Janeiro (Universidade Federal do Rio de Janeiro–UFRJ) by the protocol number 090/11.

### Assessment of anthropometric, body composition and sexual maturity

For the assessment of body mass, a Filizola scale with 0,05kg precision was used. For measuring height, it was used a 1mm-resolution metric stadiometer, where the individual was measured while standing barefoot. The body fat percentage, lean mass, fat free mass and fat tissue mass was calculated through GE-Healthcare’s Dual-energy X-ray absorptiometry (DXA) using Encore 2008 version 12.30 software. Sexual maturity was assessed through self-examination based on the Tanner scale[26] using real life photographs and drawings as standards.

### Assessment of basal metabolic rate (BMR)

Examinations were conducted by indirect calorimetry, following the FAO/WHO/UNU[8] protocol, at the Nutritional Evaluation Laboratory LANUTRI at the Nutrition Institute of UFRJ. As an exclusion criteria, one month before the exam athletes were instructed not to use any thermogenic food or supplement, stimulants, sleep or appetite inhibitor, analgesic or any other substance known to interfere in the basal metabolic rate in this period.

In addition, one day before the test, the athletes were instructed to have a 12 hour fast and not to join in any physical practice at the day before the examination. To avoid physical exertion on the day of the examination, the athletes were taken home by a driver. Upon athletes’ arrival, individual interviews were conducted in order to verify adhesion to guidelines of BMR measuring protocol. For the IC examinations, the Vmax Encore 29 System (VIASYS Healthcare Inc., Yorba Linda, CA) calorimeter was used. The examinations took place invariably in the morning, in thermoneutral environment with controlled pressure, humidity and temperature, with the patient supine, yet awake, being examined a maximum of two subjects per day. The consumption of oxygen and the production of carbon dioxide were obtained by canopy and continuously checked for 30 minutes. For the calculation, the initial 10 minutes were discarded in order to guarantee higher data homogeneity[27]. The values of VO_2_ and VCO_2_, were used at the equation proposed by Weir[28], considered the standard method[29].

### Adherence to BMR protocol

In order to ensure athletes were fit for all inclusion criteria, as well as no exclusion criteria, they were interviewed along one month prior to and at the day of the exams.

To evaluate athlete’s adherence to the food consumption criteria for the BMR assessment, 24-hour food recall (24hFR) and food frequency questionnaires (FFQ) were applied. The 24hFR was used to evaluate the athlete's routine on the previous day and the data from the FFQ were used to control food intake over the last month before IC exam. In these questionnaires, we included questions to assure they had been fasting for 12 hours and haven’t taken any food or supplement that could possibly alter the results of IC, such as caffeine, taurine, glucoronolactone, Guarana, Ginkgo biloba, Carnitine, Panax ginseng, Green Tea, Yerba Mate, among others (International Society of Sports Nutrition position stand: energy drinks).

An adapted version of the questionnaire proposed by Bouchard et al.[30] was used to assess daily activities of the athletes (e.g.: hours of sleep, timing of meals, time spent in school–with special detailing of physical education classes, when applicable–studying, watching television, using a computer). Thereby, it was possible to evaluate if they practiced 3 hours of pentathlon a day, 6 days a week, and also if they did not train for 24 hours prior to the IC test.

Along with the consumption questionnaires, the use of pharmacons, as well as the type, aim, posology and prescriber, were also investigated. The forms were self-reported or registered by their respective physical trainers. All of the athletes adhered to the BMR protocol, hence none was excluded.

### Predictive Equations

To evaluate the agreement between methods we used the most cited equations reported in the literature shown in Table 1.

**Table 1:** Predictive equations given in their original unit (kcal/day).

Name	Equation
**Harris and Benedict**	
Male	66.4730 + (13.7516 x BM) + (5.0033 x H)–(6.7550 x A)
Female	655.0955 + (9.5634 x BM) + (1.84968 x H)–(4.6756 x A)
**Cunningham**	581.6 + 21.6 x FFM
**Henry and Rees**	
Male (10–18 years)	(0.084 x BM + 2.122) x 239
Female (10–18 years)	(0.047 x BM + 2.951) x 239
**FAO**	
Male (10–18 years)	17.686 x BM + 658.2
Female (10–18 years)	13.384 x BM + 692.6

BM: Body Mass in kilogram (Kg); H: Height in centimeters (cm); A: Age in years; FFM: Free Fat Mass in kilogram (Kg)

### Statistical analyses

The intraclass correlation coefficient (ICC) was calculated to measure the degree of concordance between IC and the values of predictive equations; the T- test was applied to compare the BMR mean values. The following methods were also used: the traditional Bland and Altman[31] approach and survival-agreement plots to study the degree of agreement between aforementioned methods[32]. The significance level of p<0.05 was adopted. Height, age, body mass, BMI, body fat, lean mass and fat free mass have been used in correlations with given values of BMR through indirect calorimetry. For statistical analyses, the Statistical Program for Social Sciences version 20.0 (SPSS, Chicago, IL USA) has been used.

## Results

Body composition data—expressed in mean ± standard deviation, according to gender—are presented in Table 2. All athletes were classified as eutrophic for body mass index (BMI) and adequated body fat percentage. As to sexual maturity, athletes were distributed in pre pubertal (n = 1), pubertal (n = 22) and post pubertal (n = 5).

**Table 2:** Main Characteristics of Modern Pentathlon athletes, according to the gender (n = 28); X±SD.

	Total	Male (n = 17)	Female (n = 11)
Age (years)	15 (±2)	15 (±2)	14 (±3)
Body Weight (kg)	55.95 (±8.28)	58.52 (±8.69)	52.60 (±6.69)
Height (cm)	166.00 (±8)	169.00 (±7)	161.00 (±6)
BMI (kg/m^2^)	20.30 (±1.54)	20.38 (±1.66)	20.19 (±1.44)
Fat Body (%)	18.33 (±6.85)	14.24 (±1.97)	23.66 (±7.33)
Fat Free Mass (%)	80.89 (±8.14)	84.96 (±3.77)	74.60 (±9.20)

Sample’s stratification by sexual maturity was not possible, since it was a homogeneous sample. Using the ICC analyses, IC shows a significant positive correlation (p<0.01) with fat free mass (0.745), height (0.705), and weight (0.676), as well as a negative correlation with body fat percentage (-0.502; p<0,05).

The BMR data from the studied sample, measured by IC and estimated by equations, is presented in Table 3. Data analysis from all the athletes studied, demonstrated that only FAO’s equation differs significantly from the average values assessed by IC (p<0.01), which overestimated the BMR. The gender specific analysis, exhibited in Table 4, demonstrated that among males FAO and HR overestimated BMR when compared to IC. The BMR results among female athletes showed that all predictive equations achieved a difference lower than 2% (p>0.05), with the exception of the HR (p = 0.03), that underestimated the BMR.

**Table 3:** Basal metabolic rate (Kcal.day-1) of the studied sample, measured (IC) and estimated by the equations (FAO, HB, HR and CUN), (n = 28).

	Total				Male	Female
	Mean ± SD	Median	Minimum	Maximum	Mean ± SD	Mean ± SD
Indirect Calorimetry	1479.53 ±204.43	1495.30	1243.59	2039.63	1559.10 ±202.80	1356.57 ±140.11
FAO/WHO/UNU	1560.21 ±202.45	1507.13	1230.64	1880.30	1679 ±151.90	1375.67 ±109.73
Harris & Benedict	1513.57 ±175.29	1459.12	1258.22	1793.08	1609.62 ±148.79	1365.61 ±88.73
Henry & Rees	1514.17 ±240.84	1468.80	1156.86	1894.41	1666.61 ±172.42	1278.58 ±92.09
Cunningham	1487.49 ±212.00	1496.8	1161.85	1814.48	1579.97 ±171.09	1344.31 ±193.45

**Table 4:** Statistical parameters from comparison between BMR values obtained by indirect calorimetry in relation to different predictive equations, according to gender.

	Total (n = 28)		Male (n = 17)		Female (n = 11)	
	Test T	ICC	Test T	ICC	Test T	ICC
FAO/WHO/UNU (FAO)	0.009*	0.673	0.009*	0.466	0.553	0.677
Harris & Benedict (HB)	0.213	0.720	0.225	0.565	0.766	0.667
Henry & Rees (HR)	0.300	0.697	0.021*	0.506	0.030*	0.531
Cunningham (CUN)	0.781	0.753	0.599	0.644	0.765	0.712

*p<0,05

Test T: value of *p*, ICC: Intraclass correlation index

**Fig 1:**
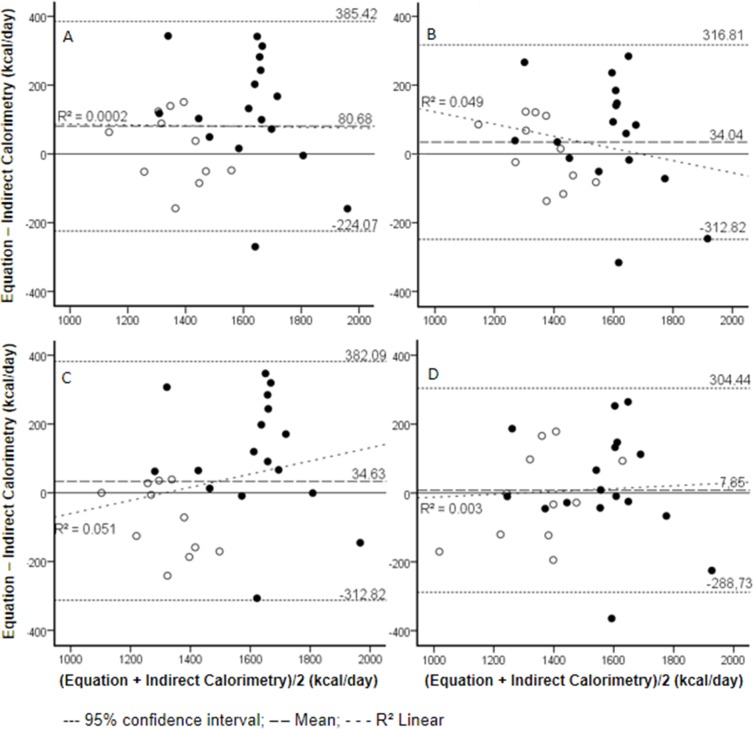
Bland and Altman scatter plots for male and female. (A) FAO; (B) Harris and Benedict; (C) Henry and Rees and (D) Cunningham. The dashed lines represent the mean bias and 95% limits of agreement of the raw data. Filled dots represent males and opened dots represent female individuals.

When BMR was analysed considering the individual values obtained from IC and the equations in Bland and Altman[31] scatter plots, on Fig 1, differences up to 350 Kcal.day^-1^ were found among the tested methods.

**Fig 2:**
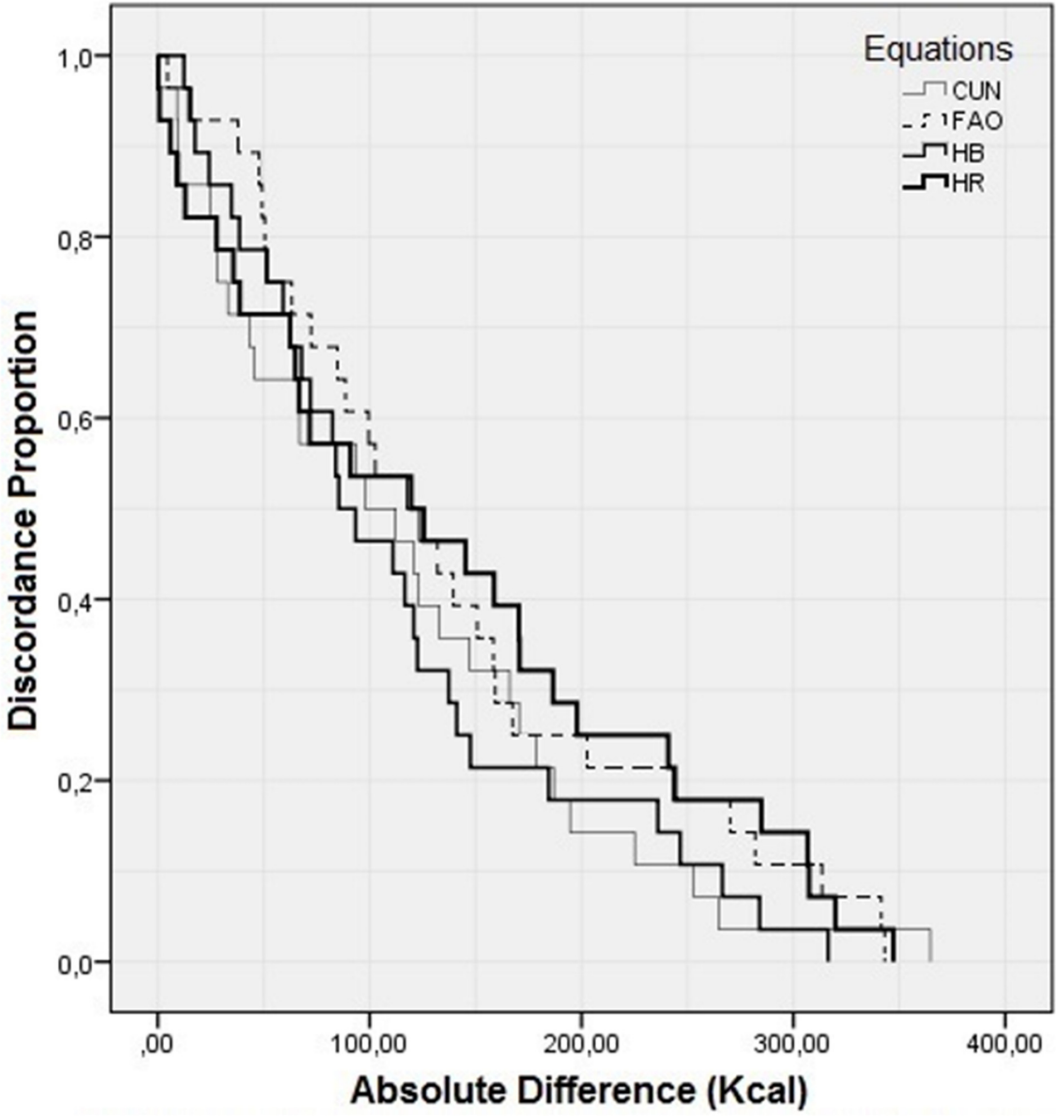
Survival-agreement plot for indirect calorimetry and predictive equations.

The agreement-survival plotting (Fig 2) confirms the findings of the Bland and Altman[21] graphs, regarding the lack of agreement between the methods when evaluating the BMR at the individual level. In this analysis values were presented in modulus, not taking positive or negative signs into account.

## Discussion

In the literature, there is still no consensus on the accuracy of BMR predictive equations in diverse populations or specific groups, such as athletes. Several studies have suggested that currently validated prediction equations overestimate BMR in relation to the value obtained by IC[33–37]. On the other hand, with athletes, the results from some studies show an underestimation of energy expenditure[38–40].

This study has been conducted to assess the applicability of BMR prediction equations—commonly used in the general population—for the assessment of adolescent modern pentathlon athletes daily energy expenditure, using (IC) as gold standard, and to correlate anthropometric and body composition variables to BMR values obtained by the IC.

The sample of adolescent athletes examined in this study presented anthropometric features similar to other studies conducted with sedentary Brazilian adolescents[41,42]. Despite the fact that these adolescents are involved in oriented physical training activities and compete in official events of the sport in question, their body features are still in development, and therefore are similar to the sedentary population.

Although only FAO differ significantly from IC when evaluated data in average, the results analyses according to Bland and Altman[31] and to survival-agreement[22] showed that there is no strong concordance between the equations and the standard IC method.

Frankenfield et al.[43] in a literature review, showed a good example of what was observed in the present study. Considering that 50% of the studied sample obtained 500 kcal under the mean and that the other 50% obtained 500 kcal above the mean, it becomes clear that the predictive equations have no differences between means but a weak degree of correlation.

In accordance with Bland and Altman[31], the ICC results are not significant although the BMR means did not differ between the equations. Depending on which equation is being used to assess BMR, there may be a difference from the results obtained from IC. When it comes to HR, this difference appears only when the sample is divided by gender, suggesting that this equation may be inadequate for energy expenditure estimations in both male and female groups. As to FAO, it may be adequate to females, but inadequate to males and to the total group. Together, this results suggest a better applicability of one or another equation based on gender.

Fonseca et al[41,42], investigating sedentary adolescent of both genders observed no significant statistical difference between IC averages and BMR predictive equations. Still, by Bland and Altman[21] and Linear Regression, the authors determined that there is no concordance between studied methods, confirming the results of the present study.

Survival-agreement plots assess the reliability of a quantitative measure, expressing the degree of disagreement (or agreement) of a measure as function of several limits of tolerance. The discordance proportion occurs exactly at absolute values of the differences between two methods, in our case, the differences between predictive equations and IC. The plot of survival-agreement (Fig 2) shows, for example, that if we consider a range of 100kcal of difference in BMR between predictive equations and IC, only 50% of the BMR values obtained with predictive equations would be fitted. For a 100% concordance between the methods, we would have to accept a difference of above 300kcal for each method when compared to IC.

According to the survival-agreement, the degree of incompatibility accepted should be less than 20%. Thus, assuming that 20% of incompatibility for the adolescent athletes present in this study, differences from 180 to 250 kcal in the BMR estimative should be accepted.

The question of what bias should be acceptable needs to be evaluated in a practical point of view, and cannot be answered using only population statistical measurements[44]. In theory an under- or overestimation of 10% in the prediction of BMR seems insignificant for a population, but it can be more relevant in an individual approach, especially in this study, where 10% of BMR can stand for a 230 kcal difference.

For instance, if we consider a subject from this study’s sample, whose BMR was underestimated in average 150 Kcal (10% of BMR), considering all the tested equations, his daily energy requirement would be underestimated in about 270–345 Kcal/day, depending on the PAL (ranging between 1,8 and 2,3 for trained athletes[8]). This variation can be a major interference in this individual’s dietary prescription[45] and would possibly result in a monthly loss of body weight of about 1,4kg, which could cause deficit in energy-protein recovery, increase the risk of fatigue and muscle damage among other factors that could interfere with the performance of a high level athlete. On the other hand, an overestimation would possibly result in body weight and body fat gain. Although it is not clear which body composition are ideal for pentathletes to achieve best physical performance, a high body fat percentage is not desirable, considering the demands of each involved modalities. Besides, an increase of body weight would require a bigger effort to move during exercise, increasing the athlete’s physical exhaustion.

Some studies have found weak correlations between height and BMR[34,37], disagreeing with the results found in the present study. This strong correlation indicates that the height variable should be considered in possible future studies and equations for this population. As expected, the body fat percentage showed an inverse correlation to the BMR measured through IC, which is in accordance to other studies[34,46]. The strong correlations observed between fat free mass and body weight in this study corroborate with the literature[38,47–49]. Illner et al.[47] showed a high correlation between fat free mass and BMR, in 26 eutrophic individuals. In another study, Speakman & Selman[48] reported that lean mass contributes with 50–70% of the BMR. With similar results, Johnston et al.[50] found that the fat free mass explained 62.3% of the difference in BMR values of 150 people from both genders. Although there are many articles demonstrating high correlation of fat-free mass with BMR, there are few equations that use this variable. We believe it is extremely important to include this variable not only in a future equation for this population but for all equations directed to athletes.

It is possible that the result found in this study diverges from studies that reported underestimation of BMR by predictive equations when working with athletes—except for the FAO equation—, since the lean mass of evaluated individuals was not as expressive when compared to those cited in the literature[36,37].

Although the sample in this study might be considered smaller than other studies that evaluated BMR, here we evaluated subjects that represented over 90% of modern pentathlon adolescent athletes in the country. Consequently, we need more studies with larger sample of adolescent pentathletes to furnish a strong conclusion about the accuracy of the predictive equations in this population and also to modelling a specific equation for such population.

## Conclusion

The FAO equation tends to overestimate BMR in relation to IC in the group of modern pentathlon adolescent athletes. The gender specific analysis demonstrated that, among males, FAO and HR overestimated the BMR when compared to IC, and HR differed only when used to evaluate women. Despite the fact that the other equations do not show significant difference compared with IC, analyses by ICC and T-test, Bland and Altman[21] and Survival-Agreement, make it possible to affirm that no equation was effective for this sample. To avoid errors in assessments of adolescent athletes’ energy requirements, the use of IC and individual monitoring is still required. As the evaluation of BMR is crucial for adequate TEE estimation, such errors could possibly mislead the athlete’s nutritionist prescription and impair his performance, potentially having further negative health and growing outcomes.

Due to the high cost of the IC, which makes it an inaccessible method for many sport center institutions in different countries, the most viable alternative is still the predictive equation. The most significant variables for this population were fat free mass, height and weight. We suggest that for future derivations of new equations aiming this public, these variables should be taken into account. Therefore, more studies are needed to establish a specific equation for this population.

## Supporting Information

S1 Dataset Full data of the paper. (XLSX)
